# Fine-Tuned DenseNet-169 for Breast Cancer Metastasis Prediction Using FastAI and 1-Cycle Policy

**DOI:** 10.3390/s22082988

**Published:** 2022-04-13

**Authors:** Adarsh Vulli, Parvathaneni Naga Srinivasu, Madipally Sai Krishna Sashank, Jana Shafi, Jaeyoung Choi, Muhammad Fazal Ijaz

**Affiliations:** 1 Department of Computer Science and Engineering, GITAM Institute of Technology, GITAM Deemed to be University, Visakhapatnam 530045, India; 121810312012@gitam.in (A.V.); 121810310009@gitam.in (M.S.K.S.); 2 Department of Computer Science and Engineering-AIML, VNR Vignana Jyothi Institute of Engineering and Technology, Hyderabad 500090, India; parvathanenins@gmail.com; 3 Department of Computer Science, College of Arts and Science, Prince Sattam Bin Abdul Aziz University, Wadi Ad-Dawasir 11991, Saudi Arabia; j.jana@psau.edu.sa; 4 School of Computing, Gachon University, Seongnam-si 13120, Korea; 5 Department of Intelligent Mechatronics Engineering, Sejong University, Seoul 05006, Korea

**Keywords:** DenseNet-169, computational histopathology, cancer, whole-slide images, lymph nodes, FastAI, 1-cycle policy, diagnostic odds ratio

## Abstract

Lymph node metastasis in breast cancer may be accurately predicted using a DenseNet-169 model. However, the current system for identifying metastases in a lymph node is manual and tedious. A pathologist well-versed with the process of detection and characterization of lymph nodes goes through hours investigating histological slides. Furthermore, because of the massive size of most whole-slide images (WSI), it is wise to divide a slide into batches of small image patches and apply methods independently on each patch. The present work introduces a novel method for the automated diagnosis and detection of metastases from whole slide images using the Fast AI framework and the 1-cycle policy. Additionally, it compares this new approach to previous methods. The proposed model has surpassed other state-of-art methods with more than 97.4% accuracy. In addition, a mobile application is developed for prompt and quick response. It collects user information and models to diagnose metastases present in the early stages of cancer. These results indicate that the suggested model may assist general practitioners in accurately analyzing breast cancer situations, hence preventing future complications and mortality. With digital image processing, histopathologic interpretation and diagnostic accuracy have improved considerably.

## 1. Introduction

Breast cancer is a severe illness that will impact one in nine women throughout their lifetime. It is observed from the surveys that 1 in 32 women may die from breast cancer. In 2018, breast cancer was expected to account for approximately 1 in 4 cases of cancer identified in women and amounted to the second-biggest category of new cancer cases. Breast cancer is the most significant risk factor for cancer in women and the seventeenth most important cause of mortality worldwide. It is the most prevalent kind of malignancy among women aged 15–49 years and the third most frequent malignancy in women aged 50–59 years [1].

Despite significant breakthroughs in understanding diseases and implementing treatment options, breast cancer remains the most frequently diagnosed cancer globally. Furthermore, it is the second leading reason behind deaths related to cancer in women [2,3,4,5]. Cancers of the lymphatic and blood vessels, which ultimately spread to distant parts of the body, are the leading cause of breast cancer mortality from metastatic (spread throughout the body) sources of breast cancer (MBCs) [6,7]. Even being diagnosed with benign breast cancer, it is expected that 10 to 50% of patients would ultimately develop metastases [8,9]. Metastasis rate and location are variables that depend on the underlying tumor subtype. As a result, prognosis, precise diagnosis, and treatment for MBCs remain difficult.

A lymph node examination is essential for diagnosing cancer and determining suitable therapy choices. Multiple lymph node levels are involved in assessing the prognosis, and appropriate staging requires meticulous examination of lymph node health. On the other hand, manually screening several slides may be tiresome and challenging for the pathologist, and individuals must undergo multiple scans for precise assessment, which is hazardous. As a result, advances in automated tissue categorization utilize machine learning techniques that precisely identify metastases over lymph node tissue [10]. The area of computer-aided diagnosis and digital pathology has progressed dramatically over the previous decade. Slide digitalization is now possible, with better resolution and spatial picture quality similar to traditional light microscopy. Digital pathology minimizes human-prone errors. Digitized WSIs provide many benefits, including viewing samples remotely for consultation and remote analysis of the slide samples, decreasing the requirement for on-site expertise [11]. 

Machine intelligence (MI) has transformed oncological research in recent years. Numerous studies have demonstrated that MI can correctly classify tissue samples as benign or malignant, particularly on hematoxylin and eosin (H&E) spattered slides. It has been shown that intelligent models, specifically image interpretation using convolutional neural networks (CNN), can accurately discriminate between malignant and benign from the images in prostate biopsies. Additionally, computer-controlled Gleason grading attained a comparable level of accuracy to that of specialized pathologists performing Gleason grading. Recent research has demonstrated that CNNs can also detect changes in protein expression and genetic mutations on H&E slides of cancer samples from various cancer types, such as prostate cancer, breast cancer, and liver cancer [12]. These findings imply that genetic mutations alter cell signaling and interaction, resulting in a change in morphology detectable by CNNs. Thus, MI can identify oncologically significant patterns from H&E images and use those sequences to predict oncological findings, such as metastasis risk or tumor recurrence.

A few studies published in peer-reviewed journals illustrate the remarkable functionality of artificially intelligent (AI) techniques through diagnostic models, like the identification of regions of interest (ROI) or perhaps the characterization and classification of types of cells, traits, or epithelial tissue [13,14]. For instance, convolutional neural networks (CNNs) have gained traction of late in detection and classification tasks because of their dependence on automated feature extraction techniques [15,16,17]. On the other hand, DL approaches have accomplished significant breakthroughs resulting in challenges and contests in image classification tasks [18]. However, although they are attractive because of their high accuracy in machine learning (ML) tasks with a massive amount of training data, there is no method yet for deciphering a DL classification model.

The motivation of the present study is to give a detailed one-stop solution for the early and automatic detection of cancer using whole slide images; early identification always has advantages. Using advanced ML libraries, we can now prevent the aggravated malignant state of cancer with minimal effort. Two terms used throughout this work are FastAI and the 1-cycle policy. FastAI is an open-source DL library [19] built using PyTorch to provide high-level DL methods for easy training of DL models. In the current study, each input image is split into smaller tiles of equivalent size, retrieving the necessary attributes/features. These features are supplied as input to the Machine Learning algorithm to faster mathematical models and predict the tumorous regions in the image. Python programming language incorporates several ready-to-use libraries for performing different MI and image processing tasks. The early detection and prognosis of the cancer type have become a necessary step in cancer research as they can help in the subsequent clinical administration of patients [20]. Even so, these days, the growth rate of cancer is increasing. Many patients die because the cause of cancer in their bodies is not recognized in time. To solve this issue, Machine Learning has risen as a promising technique for processing data with many dimensions, with increasing application in medical decisions for cancer identification and classification in histopathological images. These images include glass slide microscope images of lymph nodes stained with haematoxylin and eosin (H&E). The salient goals of the present study are as follows;

- To precisely identify the presence of metastases from 96 × 96 px digital histopathology images.
- To build a model that can precisely predict metastasis growth in early stages for better treatment.
- Fine-tuning the DenseNet-169 model by batch normalization and weight optimization strategies for a more precise outcome.
- By incorporating the 1-cycle policy and FastAI, the training rate of the model would tremendously increase and assist in faster convergence towards the solution.

The entire manuscript is further divided into the following sections: The introduction presents generalized information about the scope of the study, motivation, and contribution. The related work presents the past research in Section 2. The Methods and Materials used in the current study are presented in Section 3, and the Proposed Method along with the architecture is discussed in Section 4. The observations and the results are discussed in Section 5, and finally, the Conclusion and Future Scope are presented in Section 6. 

## 2. Literature Review

With the advancement of technology, scientists and researchers worldwide have put in a lot of effort to develop robust frameworks and methodologies for the early and effective detection of cancer using image processing (IP) and DL techniques. CT Scans, Ultrasound, Nuclear Imaging, and MRI scans have been used extensively for cancer detection. However, none of those techniques have given a highly accurate cancer prediction. Therefore, researchers have shown more interest in histopathological WSI for cancer detection in the past few years. The application of CNNs to detect different ailments in medical images dates back to the mid-1990s [21]. Ever since then, CNN architectures have been predominantly used in the medical image analysis field for various purposes, including but not limited to neural membrane segmentation in electron microscopy [22], detection and measurement of carotid intima-media in ultrasound [23], and tumor segmentation in magnetic resonance scans [24]. In addition, histopathological whole-slide images have been previously used for scoring nuclear atypia [25], discriminating between stromal tissues and epithelial tissues [26], and breast cancer detection with deep inception and residual blocks [27].

Even though CNNs have proven effective in medical image classification, it still faces many challenges. A few of these challenges are: (1) due to the excessive deepening of cancer image classification networks, the number of training parameters increases rapidly, which leads to the threat of overfitting the model. In addition, many image samples are required to reduce the risk of overfitting, which is not always possible. Hence, data augmentation methods are used to enhance the size of the dataset and prevent overfitting the model [28]. (2) Hyperparameters are very important in the effective operation of a CNN. The learning rate is an important hyperparameter that can make or break the model. During the training process, it is necessary to adjust the learning rate of the model manually according to the progression of the training to ensure that optimal model performance is reached. However, this makes it challenging to use the real-life model by non-professional users [29]. 

Many recent studies have demonstrated that training a fine-tuned CNN rather than a new one takes substantially less time, yet these fine-tuned models outperform new models [30]. This improvement in a fine-tuned CNN happens because the weights in a fine-tuned CNN are initialized to certain values known from previous knowledge. In contrast, the weights of a CNN trained from scratch are initialized randomly, thus taking more time to converge to optimal weights. Different works in IP have ideated that the initial layers of a DL neural network learn the lower-level features of an image whereas the later layers learn the high-level features; these high-level features are specific to the learning task itself, whereas the lower-level features are more general to all images [31,32,33]. This conclusion implies that training a neural network (NN) to an abundantly available image dataset and fine-tuning the weights of the later layers in that model to fit another dataset would fetch improved and enhanced results.

In light of the fact that breast cancer is among the most frequent cancers, most samples evaluated in cancer pathology are obtained from victims of this disease [34]. Pathologists employ a few common procedures to evaluate these materials, such as immunohistochemistry (IHC) to determine the histological grade and the state of the hormone receptor. These procedures, however, may be time-consuming and limited by human mistakes and observer variability [35,36]. Tumor grade is often assessed using the Bloom-Richardson technique. This approach assesses tubule development, miotic activity, and nuclear atypia in a semi-quantitative manner [37,38]. The analysis of IHC-stained slides entails estimating the number of positive cells for a certain antigen and the level of positivity [39,40]. A major challenge in bioinformatics is developing automated cancer diagnosis systems (CAD systems) that can classify a huge corpus of images in real-time to provide an accurate cancer diagnosis while being robust enough to consider the biological variations between different patients. 

The 1-Cycle policy [20] has been mentioned widely in different domains of DL and IP. The 1-Cycle policy has developed a novel activation function [30]. The authors used the 1-Cycle policy to compare their proposed activation function (Mish) with another prominent function (Swish) and proved that it outperformed Swish when the 1-Cycle policy was applied. The authors who proposed the concept of super-convergence [31] used the 1-Cycle policy to train the Densenet model and conclusively proved that super-convergence can be achieved much faster when the 1-Cycle policy is used (20 Epochs with 1-Cycle vs. 100 Epochs without 1-Cycle). The 1-Cycle policy was also used to validate the effectiveness of a fabricated large chest X-Ray image dataset [41,42]. Multiple CNNs with and without the 1-Cycle policy were trained on the fabricated dataset. The 1-Cycle policy has also been used to develop a DL framework for the semantic segmentation of remotely sensed data [43] and much more. The idea behind 1-Cycle Policy is to use the learning rate as a regularization method to prevent overfitting when the learning rate is highest during the middle of a learning cycle. It has effectively improved the performance of different Machine Learning models, which only encouraged us to incorporate them into this work.

One of the most widely used DL frameworks/libraries, FastAI, is undoubtedly one of the most prominent paradigms in the DL world. It has been used for numerous works in DL research. Multiple DL libraries within FastAI were used to detect malicious URLs [44]. FastAI has also been used to classify cotton pests using field-based images [45]. The authors used different custom models and activation functions to classify cotton pests. For automated pavement crack segmentation [46], the authors used different entities from the FastAI library. This library was also used for detecting sarcasm using a contextual neural network [47]. From a medical image analysis perspective, the FastAI framework has been used in numerous works, from analyzing tumor microenvironments in colon adenocarcinoma whole-slide images [48] to the most recent development of COVID-ResNet, a novel and fast DL framework to classify COVID-19 from radiographs [49]. The power of the FastAI library has been used in many other past works of DL. The details of various existing models are presented in Table 1 for better comprehensibility. 

## 3. Methods and Materials

This section presents the different concepts, models, and algorithms used in this study. Essentially, this section talks about the following: FastAI deep learning framework, DenseNet-169 model architecture, the 1-Cycle Policy, and the gradient-weighted class activation mapping (Grad-CAM).

### 3.1. FastAI

FastAI is a profound DL library [19] that furnishes experts with high-level libraries and modules, giving rapid and effective best-in-class results in the domain of DL. In addition, it provides scientists with low-level features that can be blended and matched to build new models and algorithms. FastAI is the maiden DL module to give the users a relied interface to all the most utilized DL applications for time-series, computer vision, collaborative filtering, tabular data, and text. FastAI is developed around these essential design objectives: rapidly productive, easily configurable, and flexible framework. It sits on top of a set of lower-level APIs, which act as the building blocks for FastAI. In this way, a client wanting to rewrite parts of the high-level API or add specific behavior to suit their requirements does not need to understand how to use the lower-level APIs.

### 3.2. 1-Cycle Policy

The 1-cycle policy [18] improves the learning rate from a predefined value to a high learning rate and then from that high value to some minimal learning rate, a lot lower than the predefined learning rate. This strategy was first depicted in Super-Convergence [33]. The 1-cycle learning rate strategy changes the learning rate after each training batch. Therefore, the learning rate step must be called after a single batch has been utilized for training. A cycle can be described in one of the two following ways:

An incentive for total steps is mentioned explicitly.

A few epochs and a few steps for every epoch (*steps_per_epoch*) are given. As shown in the following Equation, the instances of incremental steps are evaluated in this situation.

(1) total_steps=epochs×steps_per_epoch

A value must be offered for the total steps, or a discount must be given for the epochs and the *steps_per_epoch*. The default behavior of this scheduler follows the FastAI-defined execution of the 1-cycle policy. Naturally, it is useful to shift the learning rate towards a higher magnitude to help escape saddle points. If the saddle point is a plateau, the lower learning rates will probably not be easily escapable.

### 3.3. Gradient-Weighted Class Activation Mapping (GRAD-CAM)

Gradient-weighted class activation mapping (Grad-CAM) [65] uses class-specific gradients input reaching the final convolutional layer of a CNN to generate an approximate localization map of the image’s key areas. Grad-CAM is a class-discriminative localization approach that generates visual explanations to make any CNN model more understandable. Grad-CAM is a generalization of Class Activation Mapping; it requires no retraining and applies to any CNN-based model. It fabricates Grad-CAM (and/or Guided Grad-CAM) with visual explanations to more readily comprehend image captioning, visible question answering (VQA) models, and image classification. Utilizing Grad-CAM, we can visualize where our model is looking, verifying that it focuses on the appropriate patterns in the picture and activates around those patterns. Assuming the model is not activating around the appropriate patterns in the picture, it could be one of the listed reasons. The proposed model has not learned the correct insights from the training dataset.

- Our training method must be looked at
- We might have to collect additional data
- Maybe the model is not prepared yet for deployment

## 4. Proposed Method

**Figure 1:**
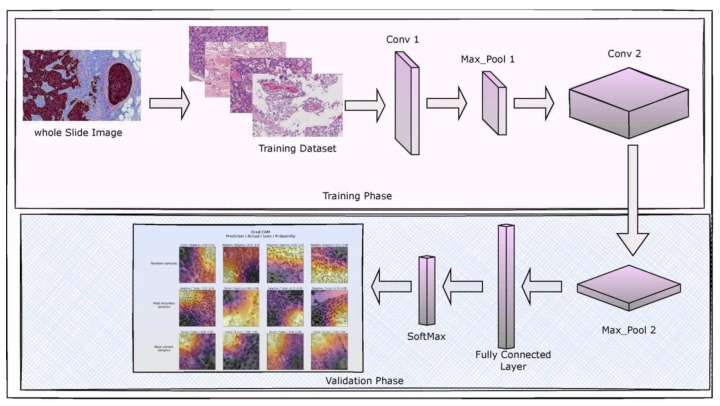
Image denoting the block diagram of the proposed model.

This section gives an overview of the architecture of the proposed model, the dataset used, weight assignment and optimization, the hyperparameters used to validate the model’s performance and the implementation environment. The initial stage of the proposed DenseNet-169 encompasses the data pre-processing task. Upon performing the data pre-processing, the images are divided into the train and test instances, where the training data would be sent to the CNN Model. Finally, validation of Fine-tuned DenseNet-169 is performed over the test data. Figure 1 presents the block diagram that illustrates the phases involved in the current study. 

### 4.1. Data Set Description and Pre-Processing

The dataset used in this work is a filtered version of the pcam dataset. The main difference between the pcam dataset and the dataset used in this work is that all the redundant pictures in the pcam dataset have been removed (filtered out). The pcam dataset was extracted from the Camelyon16 Challenge dataset, containing 400 H&E-stained images of sentinel lymph node zones procured and digitized at two distinct centers utilizing a 40× objective. The pcam’s dataset utilizes 10× under-sampling to expand the field of view, with a pixel resolution of 2.43 microns. From the data description, the positive and negative data instances are equally balanced for the training and testing portions of the dataset. The training dataset has an approximate 60–40 negative-positive distribution. A positive label implies at least one pixel of tumorous tissue in the picture’s central portion (32 × 32 Th px). Tumorous tissue in the external area of this patch does not impact the label. This means that a negatively labeled image might have metastases on the outside. As a result, cropping the photographs to the center section might be a suitable option. The pcam dataset is described in full in Table 2.

**Figure 2:**
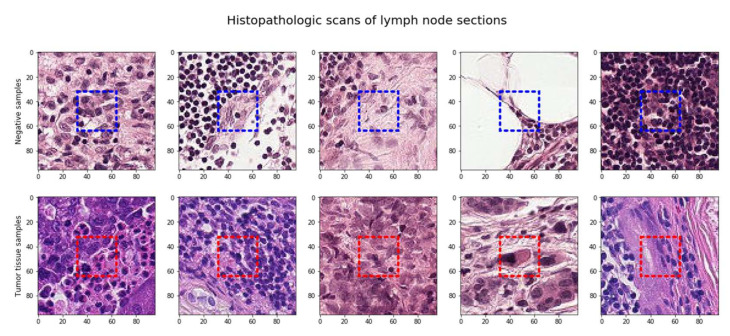
Random sampling of the dataset.

The sample raw images that are part of the pcam dataset are presented in Figure 2. The raw images are then processed to focus on the region of interest for better insight into the features and precise prediction using the data augmentation technique. 

### 4.2. Data Pre-Processing and Augmentation

**Figure 3:**
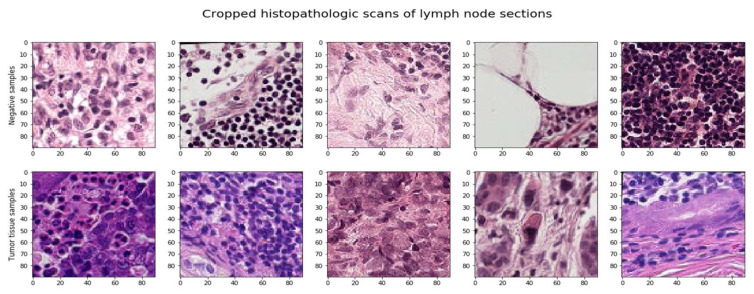
Cropped histopathological scan image.

The image label is influenced only by the center region (32 × 32 px), so it would make sense to crop our data to that region only. However, there is always the loss of valuable information around the image. Data augmentation, data regularization, and simpler model designs might be utilized to prevent overfitting the model. The approaches for picture augmentation were immediately included in the image loader function. Additionally, test time augmentation (TTA) improved the outcomes and average forecasts throughout the testing phase, as seen in Figure 3.

Data Augmentation is used in image processing tasks to create new examples from the existing training data. The model can learn from an extended range of examples and generalize well to different possible orientations of the input images. Adding to that, a small dataset size usually leads to the overfitting of the model. Hence, creating more data using data augmentation introduces variety to the input data and helps avoid the overfitting of the model. The data augmentation techniques used in the current work include random rotation, random crop, random flip, and random light, which are elaborated on in the current section.

#### 4.2.1. Random Rotation

Random rotation encompasses rotating the training images to different angles so that the essential meaning of the image does not change, however, it gives a new point of view to the model being trained. When a model may be utilized in a non-fixed location, rotating the picture (e.g., through a mobile interface) is crucial. Rotating a picture may be problematic since graphical glitches on the image’s edges might be problematic.

#### 4.2.2. Radom Crop

Cropping is essentially selecting a region of the image and saving it as a new training instance. This cropping region might be chosen randomly or based on some strategy. Cropping also involves making an image square by expanding existing dimensions to suit a square or maintaining the existing aspect ratio and adding additional pixels to fit in the newly produced empty spaces.

#### 4.2.3. Random Flip

Randomly flipping a picture around its x- or y-axis (while maintaining the image’s core structure and meaning) drives our model to realize that an item does not always have to be interpreted either left to right or up to down.

#### 4.2.4. Random Lighting (Brightness, Contrast)

**Figure 4:**
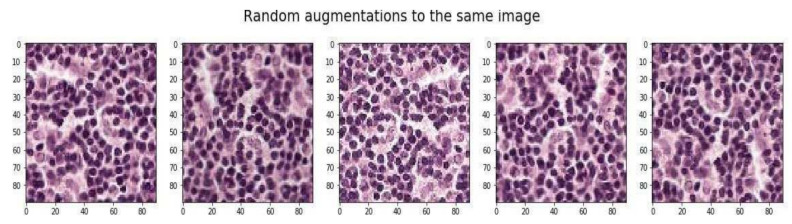
Random augmentation of cropped scan images.

If a model must perform in various lighting conditions, adjusting image intensity to be arbitrarily brighter and darker is most beneficial. Changing the intensity to match the situations the model will encounter in production, instead of the pictures provided for training, aids with generalization. The random augmentation of WSI is shown in Figure 4.

### 4.3. Layered Architecture

**Figure 5:**
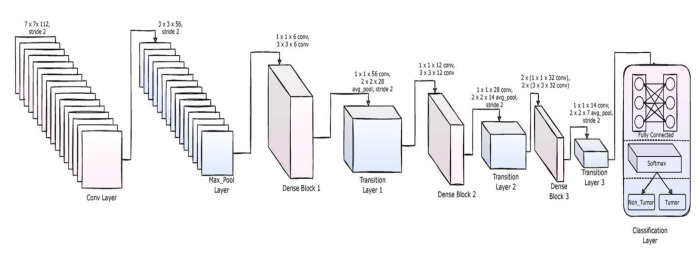
The architecture of DenseNet-169 used to implement the proposed method.

DenseNet-169 is one of the architectures of the DenseNet family with 169 layers and is a widely used architecture for DL classification tasks. It has far less trainable parameters when compared to its fellow DenseNet architectures with fewer layers. DenseNet-169 and the other DenseNet architectures have the ability to overcome the vanishing gradient problem, have a strong feature propagation strategy, minimize the number of trainable parameters, and encourage the reuse of features, thus making them a family of very reliable DL architectures. DenseNet models can be found in Tensorflow (Keras) and PyTorandes. The layered architecture of the DenseNet-169 used in the current study is presented in Figure 5.

The architecture involves convolutional layers, maxpool layers, dense layers (fully connected layers), and transition layers. The model uses the ReLU activation function throughout the architecture and uses SoftMax activation for the final layer. The convolutional layers extract the features in the image, and the maxpool layers reduce the dimensionality of their inputs. The fully connected layers follow the flatten layer, which acts as an artificial neural network with a single array input coming from the flatten layer. The details of the layered architecture are depicted in Table 3.

***Convolution Layer:*** A convolutional layer, in basic words, applies a filter to an input, resulting in the activation. When the filter is applied repeatedly to an input, the result is a feature map representing the intensity of the discovered features at different positions in the input. Once a feature map is created using multiple filters, it can be passed through activation functions such as ReLU. The filter used in a convolutional layer is smaller than the input data, and, generally, the operation performed between these two entities is a dot product. Assume a P×P square neuron component followed by a convolutional layer and a filter of size m×m, the corresponding output of the convolutional layer would be $\left(p-m+1\right)\times \left(p-m+1\right)$. To find out the non-linear input to the unit $\;x_{ij}^{l}$, the contributions from the previous layer cells must be summed up as shown in Equation (2).

(2) $$x_{ij}^{l}=\sum_{a=0}^{m-1}\sum_{b=0}^{m-1}{µ}_{ab}y_{\left(i+a\right)\left(j+b\right)}^{l-1}$$

The convolutional layer applies the assessed non-linearity as shown in Equation (3).

(3) $$y_{ij}^{l}=\lambda \left(x_{ij}^{l}\right)$$

***MaxPool Layer:*** The main purpose of using a maxpool layer in a CNN is to minimize the dimensionality of the feature map. Like a convolutional layer, the maxpool layer also runs a filter over the feature map and summarizes the features within the region covered by the pooling filter. Assume a feature map has dimensions ${\;n}_{h}\times {\;n}_{w}\times {\;n}_{c}$ that represent the height, width, and channels of the feature map, respectively. The dimensions of the feature map after applying the maximum pooling $\left(max_{p}\right)$ over the filter of size f and the stride s is defined in Equation (4)

(4) $$max_{p}=\frac{\left(n_{h}-f+1\right)}{s}\times \frac{\left(n_{w}-f+1\right)}{s}\times n_{c}$$

***Dense Layer:*** A dense layer in a neural network is deeply connected with its preceding layer, i.e., each neuron of the dense layer has a connection with each neuron in its preceding layer. The neuron in the dense layer receives inputs from each neuron in its previous layer and performs a matrix-vector multiplication. Following is the standard formula for a matrix-vector multiplication as shown in Equation (5)

(5) $$M\;\cdot \;\lambda =\begin{array}{cccc} m_{11} & m_{12}\;\;\ldots \ldots \ldots & m_{1y} & p_{1}\\ m_{21} & m_{22}\;\;\ldots \ldots \ldots & m_{2n} & p_{2}\\ \vdots & \vdots \;\;\;\;\;\;\;\;\;\;\;\;\;\;\;\;\;\;\;\; & \vdots & \vdots \\ \vdots & \vdots \;\;\;\;\;\;\;\;\;\;\;\;\;\;\;\;\;\;\;\; & \vdots & \vdots \\ m_{x1} & m_{x2}\;\;\;\ldots \ldots \ldots & m_{xy} & p_{y} \end{array}$$

From the above Equation, the variable M denotes a matrix of dimensions x×y, and other matrix p whose dimensions are 1×y. The variable λ matrix is the parameters (trained) of the preceding layer, and these can be updated using backpropagation during the training process. Using backpropagation, the weights associated with the layer ly identified by $\omega^{ly}$ and bias identified by the variable $B^{ly}$ of the neural network are adjusted using Equations (6) and (7) over the learning rate that is identified by α.

(6) $$\omega^{ly}=\omega^{ly}-\alpha \times d\omega^{ly}$$

(7) $${Β}^{ly}={Β}^{ly}-\alpha \times d{Β}^{ly}$$

The dω and db are calculated based on a chain rule (from the output layer through the hidden layers to the input layer). dω and db are the partial derivatives of the loss function of ω and b. dω and db are calculated using Equations (8)–(11).

(8) $$d\omega^{ly}=\frac{\partial L}{\partial \omega^{ly}}=\frac{1}{n}\;dZ^{ly}A^{\left[ly-1\right]T}$$

(9) $$d{Β}^{ly}=\frac{\partial L}{\partial {Β}^{ly}}=\frac{1}{n}\;\sum_{i=1}^{n}dZ^{ly\left(i\right)}\;$$

(10) $$dA^{ly-1}=\frac{\partial L}{\partial A^{ly-1}}=W^{ly^{T}}dZ^{ly}$$

(11) $$dZ^{ly}=dA^{ly}\times g^{\prime }\left(Z^{ly}\right)$$

From the above equations, the variable $Z^{ly}$ is the linear activation at layer ly and $g^{\prime }\left(Z^{ly}\right)$ is differential of the non-linear function concerning $Z^{ly}$. $A^{ly}$ is the non-linear activation function at the same layer.

***Transition Layer:*** A transition layer is used in a CNN to reduce the complexity of the model. A typical transition layer minimizes the sum of channels by using a 1 × 1 convolutional layer and decreases the width and height of the input by half using a filter with stride 2.

***SoftMax Activation Function:*** The softmax activation function is a standard non-linear activation used widely for classification problems in deep learning architectures. The general form of a non-linear activation function is defined in Equation (12), with weight identified by the variable w, and the variable b represents the bias over an input vector x.

(12) $$y=f\left(w\times x+b\right)$$

The softmax function is engaged with the output layer of a convolutional neural network when predicting the probabilities of each output class. By definition, the softmax function outputs one value for every neuron in the output layer. The output by each such neuron in the output layer is the likelihood (or probability) of that node being the output. The softmax function is defined over the softmax function Θ, applied to the input $\upsilon_{i}$ concerning the input vector’s exponential function identified by $e^{\upsilon_{i}}$ and the output vector exponent function identified by $e^{\upsilon_{o}}$ with m instances as defined in Equation (13)

(13) $$\Theta {\left(z\right)}_{x}=\frac{e^{v_{i}}}{\sum_{y=1}^{m}e^{v_{o}}}$$

With softmax as the activation function, the loss function used in this work is the binary cross-entropy loss function. Conventionally, binary cross-entropy is used while dealing with binary classification problems. Equations (14) and (15) depict the binary cross-entropy loss function, for a network of n layers.

(14) $$K\left(W,\;b\right)=\frac{1}{n}\sum_{i=1}^{n}L\left({â}^{\left(i\right)},\;a^{\left(i\right)}\right)$$

(15) $$L\left(â,\;a\right)=-\left(a\times \log â+\left(1-a\right)\times \log\left(1-â\right)\right)$$

where the variable a represents the output class 1 and $\left(1-a\right)$ denotes the output class 0. $\hat{a}$ denotes the probability of the output class 1 and the $\left(1-â\right)$ denotes the probability of the outcome associated with class 0.

### 4.4. Initial Feature Weights Assignments

The primary purpose behind optimal weight initialization is to prevent the explosion of layer activation functions or the vanishing gradient problem during forwarding propagation in a feedforward network. If one of these two issues occurs, then the gradient loss will either be too small or too large, and the network will take excessive time to converge even if it can do so. If the network weights are initialized optimally/properly, then the task of loss function optimization will be accomplished in minimal time; otherwise, converging to the minimum using the gradient descent approach will be highly impractical. Since weight initialization is significant for neural network training in DL, different techniques can initialize weights for a neural network. The most widely used technique is the random weight initialization technique.

One of the best practices while initializing weights is using leaky ReLU or ReLU as the activation function. They are resistant to the exploding or vanishing gradient problems, and leaky ReLU never has a zero gradient, ensuring continual training. Another good practice is using heuristics to initialize the weights in a network. While using the ReLU activation function, the He et al. [66] heuristic is used. In this technique, the randomly initialized weights identified by the variable $I_{w^{s}}$ is multiplied by the weight matrix $w^{s}$ over a layer of size layer s and the corresponding bias associated with the layer. If the size is equivalent to 1, the weight matrix of the dimension size of the layer identified as ($size_{ly}\times size_{ly-1})\;$ is defined using Equations (16) and (17).

(16) $$I_{\omega^{s}}=\sqrt{\frac{2}{size^{\left[ly-1\right]}}}$$

(17) $$\omega^{ly}=np.randn\left(size_{ly},\;size_{ly-1}\right)\times np\cdot sqrt\left(2/size\_ly-1\right)\;$$

### 4.5. Weight Optimization

Weight optimization involves changing the parameters such that the equation lowers according to the current instance and its nearby hits and misses at each step. It’s possible to alter a given instance’s closest neighbors by computing the closest hits and misses of every selected instance at any given time. For k-nearest neighbors, the method iterates m times, resulting in Equation (18).

(18) $$w_{d}=w_{d}-\frac{1}{k\times m}\;\left(\sum_{a=1}^{k}\rho^{R}\left(e_{d}^{b},\;e_{d_{hit}}^{b_{a}}\right)+\sum_{a=1}^{k}\rho^{R}\;\left(e_{q}^{b},\;e_{q_{miss}}^{b_{a}}\right)\right)$$

where $e_{d_{hit}}^{b_{a}}$ denotes the *d*th feature value of the nearest hit of the instance b and $e_{q_{miss}}^{b_{a}}$ represents the *d*th feature value of the *a*th the nearest miss of the instance b. The variable $\rho^{R}$ denotes the similarity among the instances [67]. Weight optimization can be achieved from Equation (19), over gradient descent approach having a constant learning rate of $\frac{1}{k\times m}$.

(19) $$j_{w}^{R}=\sum_{a=1}^{k}\sum_{d=1}^{Q}w_{d}\rho^{R}\;\left(e_{d}^{b},\;e_{d_{hit}}^{b_{a}}\right)-\sum_{a=1}^{k}\sum_{d=1}^{Q}w_{d}\rho^{R}\;\left(e_{q}^{b},\;e_{q_{miss}}^{b_{a}}\right)\;$$

### 4.6. Hyperparameters

**Figure 6:**
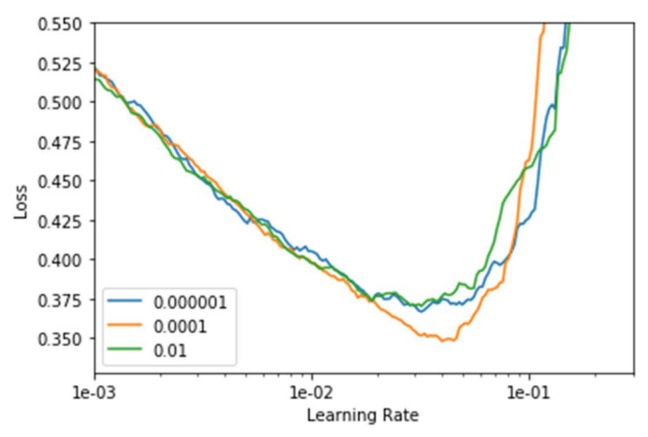
Graph representing the learning rate associated with weight decay.

The hyperparameters that are associated with the fine-tunes DenseNet-169 model, which include the learning rate and the loss associated with batch processing, are discussed in the current section. It is desirable to choose the optimal parameters for better training and testing performances and avoid underfitting and overfitting of the model. The loss and accuracy of training and testing are discussed in the current study. The ideal learning rate range is reached at the initial point of divergence of the model. Ideally, at this point, the loss must still go on decreasing when the learning rate is chosen. As for the L2 penalty of the optimizer (weight decay), the author [68] proposes to choose the largest learning rate so that it will still allow us to train at a higher learning rate over the grid-search with weight decays 0.01, 0.0001, and 0.000001 respectively as shown in Figure 6.

**Figure 7:**
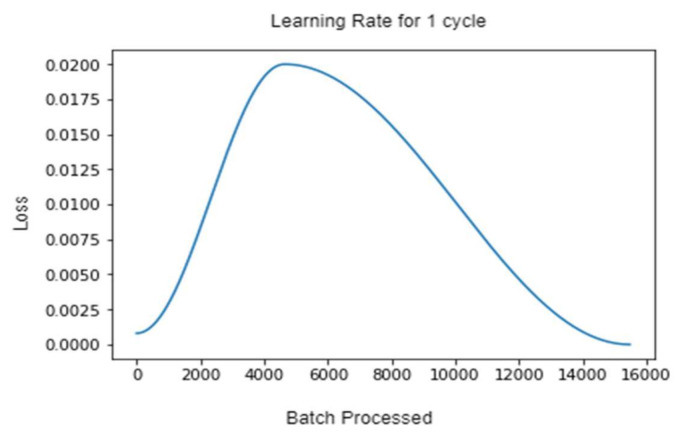
Graphs representing the learning rate for 1-Cycle policy.

The above graph in Figure 7, shows the loss of the model as the number of batches processed increases with the 1-cycle of the learning rate. As it can be seen, the training begins with a near-zero model loss, but as the instances of training batches increase, the loss increases up to around 5000 batches. After that, there’s a sharp drop in the loss, and the training in that cycle ends at a near-perfect zero loss.

**Figure 8:**
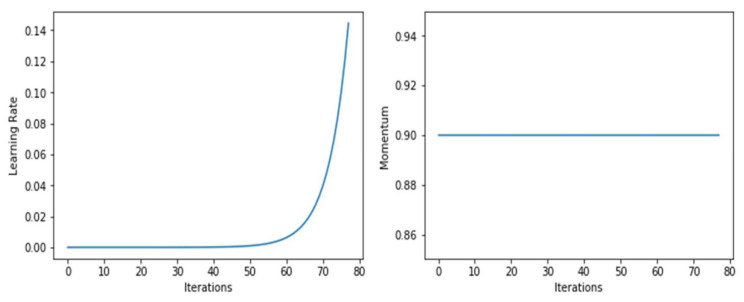
Graphs of learning rate and momentum over iterations before fine-tuning DenseNet-169.

**Figure 9:**
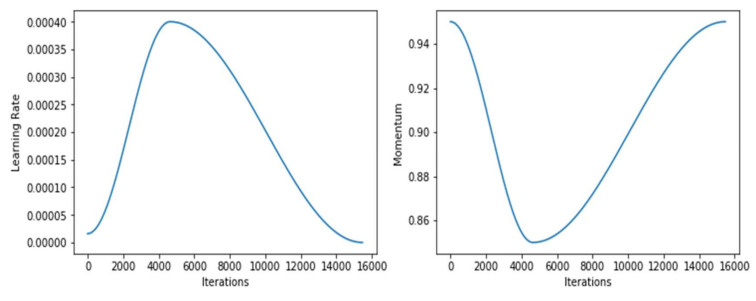
Graphs of learning rate and momentum over iterations after fine-tuning DenseNet-169.

As shown in Figure 8, the model’s momentum did not change throughout the training; it was a constant of 0.9. However, the learning rate shot up after 50 epochs. Figure 9, shows the learning rate and momentum after fine-tuning the DenseNet-169 model. 

The maximum learning rate, which is identified using the variable (max_lr), is reached in the middle of the learning process. Then it slows down again towards the conclusion of the image. Because the model cannot settle for narrow and sharp local minima, the larger rate has a regularizing impact, pushing the model toward broader and more stable ones. As we near the midpoint of our cycle, we begin to slow down our pace of learning in the hopes that we have reached a stable state. This signifies that we begin searching for the area’s smallest values.

**Figure 10:**
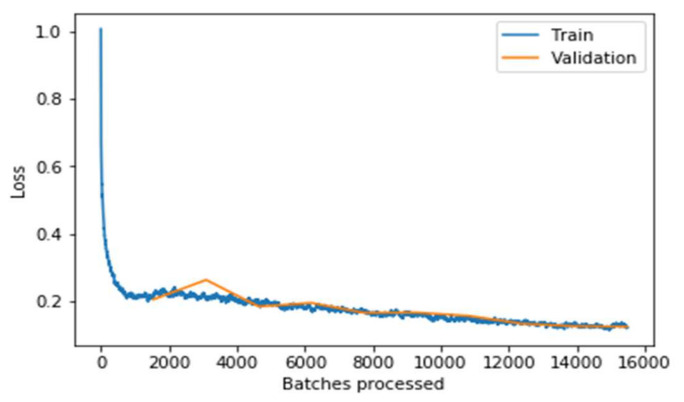
Loss associated with batches processed before fine-tuning the model.

Even before fine-tuning the model, the model performs very well during training as shown in Figure 10. On fine-tuning the model by unfreezing the bottom layers pre-trained with other data and training the model again with our cancer data, adjusting the weights of these unfrozen layers further optimizes the model. After unfreezing, we train the model with a much lower learning rate.

**Figure 11:**
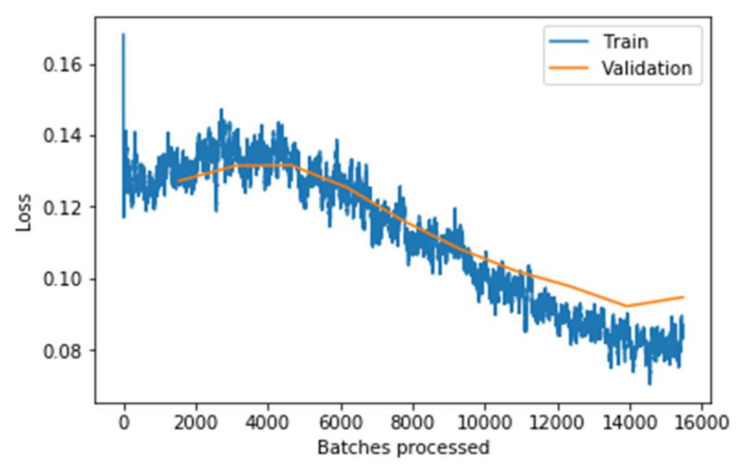
Loss associated with batches processed after fine-tuning the model.

It can be observed from the Figure 11, the graph that the training and validation losses remain pretty close throughout the multiple batches. Still, the validation loss increases slightly at the end (around the 13,000 to 16,000 batches mark). This implies that the model starts overfitting when the learning rate is low. If the model is trained further, the model will overfit more, meaning it would just memorize the features of the training set, increasing the validation performance. Still, the model will not work well on real-world data that has resulted in concluding the optimal point to stop the training. It can be concluded clearly from the above two graphs that the model performs significantly well on the data after fine-tuning the model. The model’s performance is evaluated concerning the training loss and accuracy measure. Similarly, the testing loss and accuracy with the other state-of-art models are evaluated and shown in Table 4.

It is observed that the fine-tuned DenseNet-169 model has exhibited a better performance compared to the other models, and it has proven to exhibit better performance over the conventional DenseNet-169. The feature weight optimizations have assisted in a much better way to identify the metastases in lymph nodes precisely. 

### 4.7. Implementation Environment

This experiment is carried forward over the Kaggle’s compiler (online platform) [70]. The FastAI PyTorch transfer learning frameworks build the in-depth learning technique discussed in the current study. Python programming language is used to develop the Densenet-169 DL model. Table 5 presents the environment’s specifications in which the model was trained.

## 5. Results and Discussions

In the current section, the efficacy of the F=fine-tuned DenseNet-169 model for metastases in lymph nodes is evaluated across various metrics like the sensitivity, specificity, accuracy, and F1 score. The performances are analyzed against the other state-of-art models like logistic regression (LR), neural network (NN), random forest (RF), support vector machine (SVM), CNN, and DL models like VGG-16 and ConcatNet. The proposed model has outperformed the various approaches with reasonable performance discussed in the current section. The other parameters like the receiver operator characteristic curve and the test time augmentation are discussed for better comprehensibility of the model. 

### 5.1. Confusion Matrix

**Figure 12:**
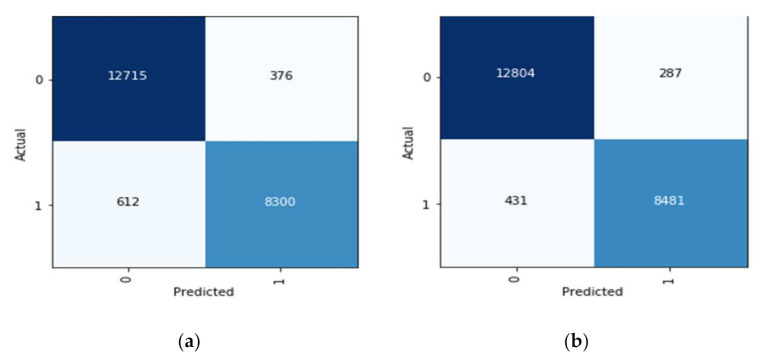
(**a**) Confusion matrix for DenseNet-169 (**b**) Confusion matrix for fine-tuned DenseNet-169.

The confusion matrix is a table that depicts the instances of true positives (TruP), true negatives (TruN), false positives (FlsP), and false negatives (FlsN) [71]. An output is called TruP when the model recognizes the instance as positive (or 1), and the actual output is positive. An output is called TruN when the model recognizes the instance as negative (or 0), and the actual output is negative. An output is called FlsP when the model recognizes the instance as positive (or 1), and the actual output is negative. An output is called FlsN when the model recognizes the instance as negative (or 0), and the actual output is positive. It can be concluded, that the more TruPs and TruNs (or fewer FlsPs and FlsNs), the more accurate the model is. The correct identification of a tumor in a slide is considered true positive, whereas no tumor in a slide is identified as true negative. Similarly, the wrong identification of a tumor is referred to as a false positive, and the wrong identification of no tumor is called a false negative. Both the DenseNet-169 and fine-tuned DenseNet-169 were trained independently, and then both were assessed based on their confusion matrices. The confusion matrices associated with DenseNet 169 are presented in Figure 12.

The fine-tuned model significantly improves over the original DenseNet-169 model from the above confusion matrices and the tables. The instances of true positives increase by 141, and the instances of true negatives increase by 89. This also implies that the instances of FPs and FNs decrease. The confusion matrix for the fine-tuned model is also used to assess the metrics such as sensitivity, specificity, and the F1-score.

For binary classification tasks in medical testing, the diagnostic odds ratio (DOR) [72] is a parameter used to assess the efficacy of a particular diagnostic test. DOR is defined as the ratio of the probability of the test being positive if the patient has the disease relative to the probability of the test giving a positive result if the patient does not have the disease. The diagnostic odds ratio (DOR) is defined mathematically as shown in Equation (20).

(20) $$\mathrm{DOR}=\frac{sensitivity\times specificity}{\left(1-sensitivity\right)\times \left(1-specificity\right)}\;\;\;\;\;\;\;\;\;i.e.,\;\;\frac{TP\times TN}{FP\times FN}$$

The DOR for Dense-Net-169 is:

$$DOR_{DenseNet169}=\frac{8300\times 12715}{376\times 612}=458.62$$

Similarly, the DOR for fine-tuned Dense-Net-169 is:

$$DOR_{FineTuned-DenseNet169}=\frac{8481\times 12804}{287\times 431}=877.88$$

The diagnostic odds ratio is greater than one for useful tests, and a higher value of DOR indicates a better performance. A DOR value of less than one indicates that the performance of the test can be improved simply by inverting the result of the test. Given this interpretation of DOR, since the DOR of DenseNet169 is 458.62 whereas the DOR of the fine-tuned model is 877.88, there is a huge jump in the DOR value, indicating a huge improvement in the performance of the model.

**Figure 13:**
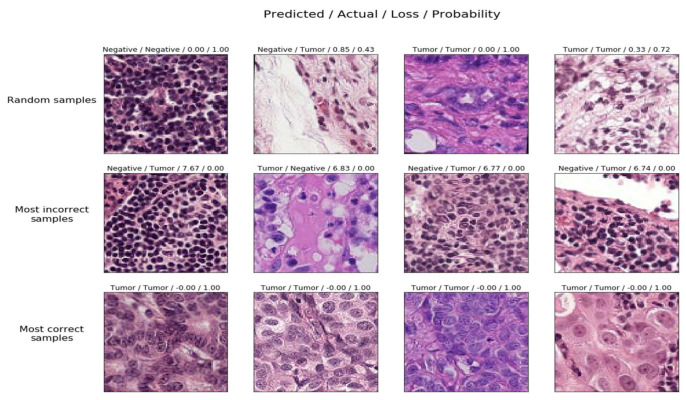
Probabilities scores associated with samples in the testing phase.

The test scores associated with each random sample in the testing phase that varies among the random samples and most incorrect samples and the correct samples and their associated probabilities to classify them either as tumors or not are presented in Figure 13 [73]. It can be interpreted from the figure the set of random samples from which the model struggles to learn. It also reveals something about the dataset, for example, the integrity of the data utilized in the training phase. A few of the observations include

- Random samples are predictions made on some random instances from the data.
- Most incorrectly labeled samples are the models predicted wrongly with a very high probability.
- The model predicted correctly with a very high probability is the most correctly labeled sample.

### 5.2. Performance Analysis with Past Studies

The performances of the fine-tuned DenseNet-169 are evaluated concerning accuracy, sensitivity, specificity, and F1-Score concerning the various state-of-art models, whose values are presented in Table 6. The values of the other models are obtained from the previous experimental studies over a similar dataset are presented. It can be analyzed from the experimental results that the performance of the fine-tuned DenseNet-169 is reasonably better than the other model considered for statistical analysis. 

### 5.3. The ROC Curve and TTA

**Figure 14:**
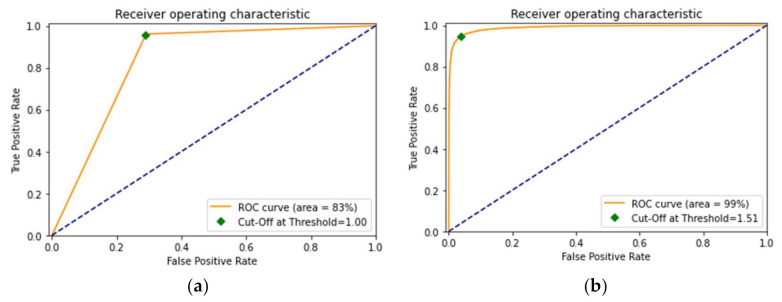
(**a**) Receiver operator characteristic (ROC) curve before fine-tuning (**b**) ROC curve after fine-tuning.

The other most predominantly used performance evaluation parameter, receiver operator characteristic (ROC), is a graphical depiction of the diagnostic ability of binary classification models. A ROC curve for a model is constructed by presenting the false positive rate of the model against the true positive rate. The ROC curve essentially depicts the dependencies among the sensitivity, i.e., true positive rate, and specificity, i.e., false positive rate. Binary classifiers which can cover the maximum area under the curve (or are closest to the top-left corner) are the best ones. Figure 14 depicts the ROC curves of the DenseNet-169 model before and after fine-tuning. As observed from the figures, the model covers 83% of the area in the graph before fine-tuning. However, its performance improves after fine-tuning as it covers 99% of the area in the whole graph, which is a pretty high area under the curve (AUC), implying that the model’s performance is optimal.

**Figure 15:**
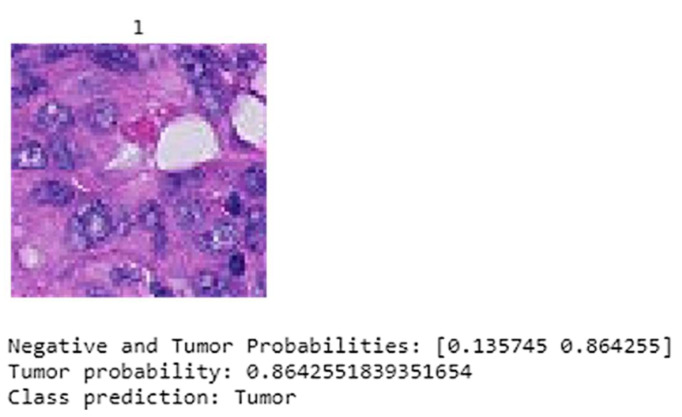
Results after applying the test time augmentation (TTA).

The test time augmentation (TTA) is considered in testing the model. The input data fed to the model is traversed through a process in which the test data is transformed with random data augmentation techniques during test time. The proposed model predicts the whole tumor image as tumorous with 86.42% probability. Figure 15 depicts the performance of the fine-tuned model after applying TTA, the 1 above the slide image denotes that the image consist of metastasis growth which is identified by the proposed model.

### 5.4. Practical Implication

The front-end interface of the mobile system may be used to deploy the proposed smart diagnosis technology for identifying the growth of metastasis. Primary in diagnosing metastasis, the mobile framework technology might assist both the patient and the doctor. Name, date of birth, gender, height, diabetes status, weight, and hypertension are among the information the users will produce initially. At the later stages, the user will provide the metastasis whole slide image for predicting the presence of metastasis. The model uses the input image and the training data to predict the abnormality over the input image. The back-end of the architecture relies on the service, like FlaskApi, which is used to integrate the iOS framework into the Kaggle. Authentication and a secure socket layer (SSL) can secure the model. 

**Figure 16:**
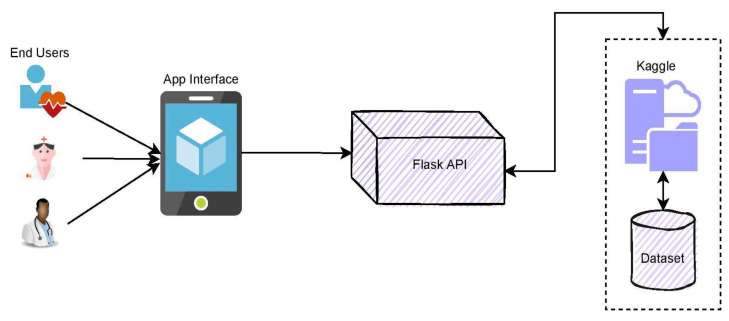
Block diagram of practical implication model.

**Figure 17:**
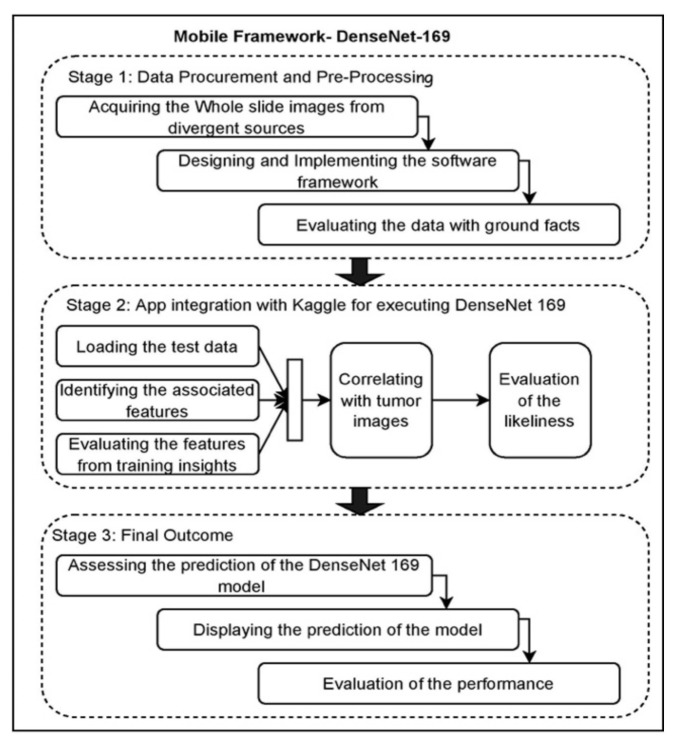
Mobile framework for app integration with fine-tuned DenseNet-169.

The block diagram for the app integration with DenseNet-169 is presented in Figure 16. The conceptual approach for practical use includes many stages in finding metastases in lymph nodes. In the initial phase, the whole slide images are procured, and they are provided to the model for the training purpose. In training the model, the ground facts are being provided over the data sample with the support of the radiologist, professionals, and practitioners. In the later stages, the testing samples are fed as the input for evaluation upon training the model. The model performs the feature identification feature processing and correlates them with the trained data, and the probabilistic measures for each sample are assessed. The resultant outcome is displayed to the end-users, and finally, the performance of the fine-tuned DenseNet-169 model is assessed and updated accordingly. Figure 17 presents the mobile framework for app integration with fine-tuned DenseNet-169.

In the proposed IntelliJ-Diagnosis mobile application, the security of the patient data is considered to be sensitive and exceptionally important to ensure the privacy of the information. To ensure the privacy of the data, the encryption of mobile data is performed. Data sent is transferred among the mobile app, and data transferred to the server is encrypted using the secure sockets layer (SSL) protocol. While data is encrypted, the asymmetric key techniques use a public key for encrypting the data and a private key that is only known to the receiver of the message [77,78]. The corresponding user data is stored in the NoSQL MongoDB. This provides users with a stronger feeling of control over their personal information, including confidentiality, privacy, and secrecy, of their healthcare data. The above-discussed technology would make the future perspective model a user-centric model with all the necessary features. 

**Figure 18:**
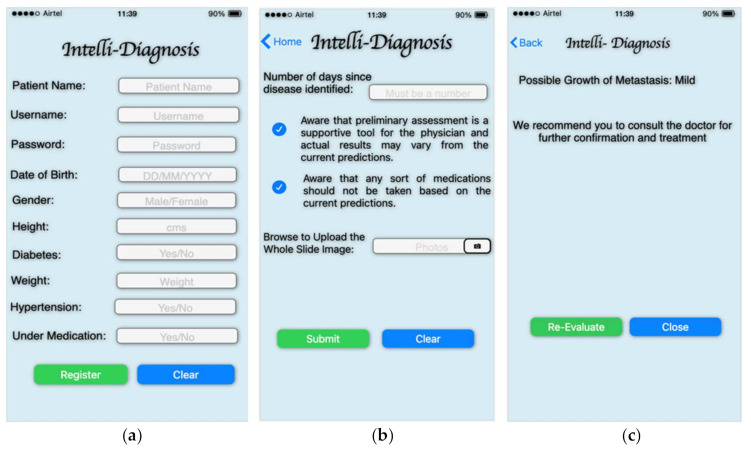
Image of user-interface of the future perspective model.

The figure above depicts the future perspective model’s user interface. Figure 18a shows the app’s registration process, Figure 18b shows the input page where the WSI data is uploaded to the model, and Figure 18c shows the outcome of the model’s prediction. The model simplifies the process of diagnosing the development of a disease. Medical professionals, radiologists, and patients alike can benefit from the predictions made by the model that would endorse the reports of the initial diagnosis.

## 6. Conclusions and Future Scope

This work aimed to facilitate the development of digital diagnosis in MBCs and explore the applicability of a novel CNN architecture on MBCs. In this paper, we proposed a fine-tuned DenseNet-169 CNN architecture to automatically diagnose the presence of cancer cells in the pathological tissue of breast cancers. The fine-tuned DenseNet-169 detects metastases from whole slide images using the FastAI framework and the 1-cycle policy. The results obtained from this research were better than any other approaches proposed earlier. The AUC-ROC of the model is 97%, whereas the accuracy of the baseline model was approximately 92%. The DL model proposed in this work can be enhanced further for other cancer data with different data augmentation techniques. A base model other than DenseNet-169 can also be used to see if a different model gives even better performance. Another area of future research could be adding noise to the region of interest and seeing how that may affect the edge cases. Therefore, it can provide an efficient, reliable, and economical alternative for medical facilities in relevant fields. In the future, the proposed DenseNet-169can be applied to diverse cancer datasets to enhance the clarity and quality of results. 

## Figures and Tables

**Table 1:** A detailed description of the various models in image processing.

Approach	Objective	Challenges of the Approach
Genetic Algorithm (GA) [50,51]	A genetic algorithm selects the beginning population at random through a probabilistic approach. It performs crossover and mutation processes concurrently until the necessary portions are reached.	The algorithm fails in producing the best output and is more time-consuming.
Fully Convolutional Residual Network (FCRN) [52]	FCRN technique employs encoder and decoder layers for image classification that use low-high level features. The feature processing is exceptionally important for the appropriate classification.	A completely Conventional Layer handles overfitting well, yet the model is complex in design and implementation. Adding batch normalization might also make the model less efficient.
Decision Tree (DT) [53,54]	Handling discrete data necessitates the usage of models based on decision trees which is a rule-based technique for predictions. It is effective in dealing with non-linear factors.	The Decision Tree model is unreliable if the input data is changed even by a small proportion, and at times DT models will lead to overfitting while training.
Bayesian Learning (BL) [55,56,57]	The Bayesian Learning technique effectively manages continuous and discrete data by avoiding the incorrect binary and multi-class classification characteristics.	The Bayesian Classifier is often an improper probabilistic model since it is unsuited for unsupervised learning applications.
Deep Neural Networks [58,59]	Deep Neural Networks may process structured and unstructured data. Models are capable of working with unlabelled data and delivering the expected results.	DNN model is a black-box decision model, and models are complex and need tremendous development efforts.
K-Nearest Neighbourhood [60]	KNN based models work on unlabelled data and classify data into different categories using feature selection and similarity matching. These models use the distance between two instances to identify their correlation.	The trained model’s accuracy is closely related to the quality of the data used to train it. In addition, the time needed to make a forecast may be much longer if the sample size is bigger.
Support Vector Machine [61,62]	Support Vector Machine is a data processing system that uses as little computing and memory as possible.	It is difficult to determine the feature-based parameters using the Support Vector Machine method, which is inefficient for noisy data.
Artificial Neural Networks [63,64]	Linear relationships between dependent and independent parameters may be easily recognized using Artificial Neural Networks, storing data across the network nodes.	Using Artificial Neural Network models is a good way to deal with a lack of knowledge of the issue. There is a good chance that the ANN will miss the spatial elements of the picture. The gradient’s diminishment and explosion are also major concerns.

**Table 2:** Dataset descript associated with pcam.

Description	Specification
Format	TIF
Input Size	96 × 96
Number of Channels	3
Bits per Channel	8
Data Type	Unsigned Char
Image Compression Approach	Jpeg

**Table 3:** DenseNet-169 layered architecture.

Layer	Kernel Size	Parameters	Tensor Size
Convolution	7 × 7 (Conv)	Stride = 2, ReLu	112 × 112
Pooling	3 × 3 (MaxPool)	Stride = 2	56 × 56
Dense-1 Layer	1 × 1 × 6 (Conv) 3 × 3 × 6 (Conv)	Dropout = 0.2	56 × 56
Transition-1 Layer	1 × 1 (Conv) 2 × 2 (AvgPool)	Stride = 2	56 × 56 28 × 28
Dense-2 block	1 × 1 × 12 (Conv) 3 × 3 × 12 (Conv)	Dropout = 0.2	28 × 28
Transition-2 Layer	1 × 1 (Conv) 2× 2 (AvgPool)	Stride = 2	28 × 28 14 × 14
Dense-3 Layer	1 × 1 × 32 (Conv) 3 × 3 × 32 (Conv)	Dropout = 0.2	14 × 14
Transition-3 Layer	1 × 1 (Conv) 2× 2 (AvgPool)	Stride = 2	14 ×14 7 × 7
Dense-4 Layer	1 × 1 × 32 (Conv) 3 × 3 × 32 (Conv)	Dropout = 0.2	7 × 7
Classification Layer	1 × 1 (Global AvgPool) 1000D (fully-connected softmax)		1 × 1

**Table 4:** The hyperparameter values are associated with various models.

	Training		Testing	
	Loss	Accuracy	Loss	Accuracy
CNN [69]	0.124	92.25	0.565	81.93
CNN + Augmentation [69]	0.164	93.82	0.621	82.13
VGG-16 [69]	0.008	99.75	0.290	79.00
ConcatNet [69]	0.108	95.90	0.435	86.23
DenseNet-169	0.152	94.61	0.411	95.57
Fine-tuned DenseNet-169	0.123	95.45	0.125	97.45

**Table 5:** Details of Implementation Environment.

Environment Details	Specifications
Operating System	Microsoft Windows 11
Processor	Intel(R) Core (TM) i7-8750H
Architecture	64-Bit
Memory Allotted	3 GB
GPU	Nvidia (TM) 1050 Ti
Language	Python
Framework	FastAI, PyTorch, DL
Libraries Used	Pandas, Numpy, cv2, Matplotlib, Scikit-learn, os

**Table 6:** Comparison of DenseNet-169 model with state-of-art models.

	Accuracy	Sensitivity	Specificity	F1-Score	Precision
Logistic regression [17]	87.0	86.4	87.6	0.87	-
NN [17]	82.8	74.4	91.0	0.81	-
NN feature subset [17]	91.3	85.7	96.8	0.91	-
Random Forest [17]	93.0	92.6	93.3	0.93	-
SVM [17]	88.3	85.9	90.6	0.88	-
CNN [61]	76.4	74.6	80.4	-	-
CNN + Augmentation [61]	78.8	80.2	81.4	-	-
VGG-16 [61]	76.5	75.3	82.6	-	-
ConcatNet [61]	84.1	82.0	87.8	-	-
Multimodal Deep Neural Networks [74]	79.4	80.0	-	-	0.875
SVM [74]	77.5	87.8	-	-	0.811
RF [74]	77.0	90.2	-	-	0.787
RF [75]	80.1	91.0	-	-	-
LR [74]	75.4	96.3	-	-	0.563
Inception V3 [76]	80.5	82.0	79.0	0.81	-
Inception-RestNet V2 [76]	82.0	80.0	85.0	0.82	-
ResNet-101 [76]	78.0	78.0	79.0	0.78	-
DenseNet-169	95.5	93.1	97.1	0.94	0.971
Fine-tuned DenseNet-169	96.7	95.2	97.8	0.96	0.978

## Data Availability

Not applicable.
